# Supplementary material for: Feedback perceptions of first year medical residents: An intervention-based survey study
Source: PLoS One. 2024 Apr 10;19(4):e0300205. doi: 10.1371/journal.pone.0300205 (PMC11006118; doi:10.1371/journal.pone.0300205)
Supplement: Attachment — Submitted filename: PONE-D-23-29943_R1_reviewer.pdf [file pone.0300205.s004.pdf]

PLOS ONE

Feedback perceptions of first year medical residents: A survey study

--Manuscript Draft--

**Manuscript Number:**	PONE-D-23-29943R1
**Article Type:**	Research Article
**Full Title:**	Feedback perceptions of first year medical residents: A survey study
**Short Title:**	Feedback perceptions of first year medical residents
**Corresponding Author:**	Rachel Cox, MD<br>Wright State University<br>Dayton, OH UNITED STATES
**Keywords:**	Trainee feedback; resident feedback; ACGME milestones; SMART goals; Deliberate Practice
**Abstract:**	Background<br>Feedback in residency is a necessity for progression toward clinical competency and is included in The Accreditation Council for Graduate Medical Education (ACGME) milestones as an essential component for accreditation.<br>Purpose<br>Our study elucidates perceptions of feedback of first-year residents and aims to identify how these perceptions change after education on building expertise through deliberate practice.<br>Methods<br>First-year internal medicine and neurology residents of a mid-sized university-affiliated residency program answered a five-question 5-point unipolar response scale questionnaire regarding feedback perceptions before and after attending a workshop about building expertise through effective feedback during residency orientation. Related-Samples Wilcoxon Signed Rank Test was applied for comparing pre- versus post-questionnaire data.<br>Results<br>Of 31 first-year residents, 29 completed the pre-questionnaire for a completion rate of 93.5%, while 24 of 31 completed the post-questionnaire for a completion rate of 77.4%. Of the five questions, three improved when comparing pre and post responses to, including the questions on confidence in the ability to procure feedback (p=<0.001), the effort put into procuring feedback (p=0.001), and frequency of seeking feedback (p=0.002). Interest in receiving feedback and the importance of feedback remained unchanged after workshop attendance.<br>Conclusion<br>Residents should be educated on building expertise through deliberate practice and how to obtain high-quality feedback, given the emphasis and essentiality of feedback within the milestone assessment system and the core competencies of ACGME. In our study, education on these topics led to significant improvement in resident perceptions of confidence in the ability to procure feedback, effort put into procuring feedback, and frequency at which feedback would be sought.
**Order of Authors:**	Rachel Cox, MD
	John Arthur, DO
	Kathryn Burtson, MD
**Response to Reviewers:**	Please find Revised Manuscript with Tracked Changes, original Manuscript, Response to Reviewers, and S1 Dataset which have been uploaded. We appreciate your time and allowing us to revise our manuscript as well as make other corrections including ensuring adherence to the formatting guidelines, making our data available, and reviewing the accuracy of our reference list. We are willing to make any further changes that are needed and we look forward to continuing our publication process with PLOS ONE.
**Additional Information:**	
**Question**	**Response**

Powered by Editorial Manager® and ProduXion Manager® from Aries Systems Corporation

**Financial Disclosure**<br><br>Enter a financial disclosure statement that describes the sources of funding for the work included in this submission. Review the submission guidelines for detailed requirements. View published research articles from *PLOS ONE* for specific examples.<br><br>This statement is required for submission and **will appear in the published article** if the submission is accepted. Please make sure it is accurate.<br><br>**Unfunded studies**<br>Enter: *The author(s) received no specific funding for this work.*<br><br>**Funded studies**<br>Enter a statement with the following details:<br>• Initials of the authors who received each award<br>• Grant numbers awarded to each author<br>• The full name of each funder<br>• URL of each funder website<br>• Did the sponsors or funders play any role in the study design, data collection and analysis, decision to publish, or preparation of the manuscript?<br>• **NO** - Include this sentence at the end of your statement: *The funders had no role in study design, data collection and analysis, decision to publish, or preparation of the manuscript.*<br>• **YES** - Specify the role(s) played.<br><br>* typeset	The author(s) received no specific funding for this work.
**Competing Interests**<br><br>Use the instructions below to enter a competing interest statement for this submission. On behalf of all authors, disclose any competing interests that could be perceived to bias this work—acknowledging all financial support and any other relevant financial or non-financial competing interests.<br><br>This statement is required for submission	The authors have declared that no competing interests exist.

and **will appear in the published article** if the submission is accepted. Please make sure it is accurate and that any funding sources listed in your Funding Information later in the submission form are also declared in your Financial Disclosure statement.<br><br>View published research articles from *PLOS ONE* for specific examples.<br><br>**NO authors have competing interests**<br><br>Enter: *The authors have declared that no competing interests exist.*<br><br>**Authors with competing interests**<br><br>Enter competing interest details beginning with this statement:<br><br>*I have read the journal's policy and the authors of this manuscript have the following competing interests: [insert competing interests here]*<br><br>* typeset	
**Ethics Statement**<br><br>Enter an ethics statement for this submission. This statement is required if the study involved:<br><br>• Human participants<br>• Human specimens or tissue<br>• Vertebrate animals or cephalopods<br>• Vertebrate embryos or tissues<br>• Field research<br><br>Write "N/A" if the submission does not require an ethics statement.<br><br>General guidance is provided below. Consult the submission guidelines for detailed instructions. **Make sure that all information entered here is included in the Methods section of the manuscript.**	This activity was reviewed by the Wright-Patterson Medical Center Human Research Protection Program (Protocol #: FWP20230001N), and determined to not constitute research following Federal Regulations and Department of Defense Instructions. The Wright-Patterson Medical Center Human Research Protection Program gave this project a non-research determination thereby waiving the requirement for informed consent.

Powered by Editorial Manager® and ProduXion Manager® from Aries Systems Corporation

**Format for specific study types**<br><br>**Human Subject Research (involving human participants and/or tissue)**<br>• Give the name of the institutional review board or ethics committee that approved the study<br>• Include the approval number and/or a statement indicating approval of this research<br>• Indicate the form of consent obtained (written/oral) or the reason that consent was not obtained (e.g. the data were analyzed anonymously)<br><br>**Animal Research (involving vertebrate animals, embryos or tissues)**<br>• Provide the name of the Institutional Animal Care and Use Committee (IACUC) or other relevant ethics board that reviewed the study protocol, and indicate whether they approved this research or granted a formal waiver of ethical approval<br>• Include an approval number if one was obtained<br>• If the study involved *non-human primates*, add *additional details* about animal welfare and steps taken to ameliorate suffering<br>• If anesthesia, euthanasia, or any kind of animal sacrifice is part of the study, include briefly which substances and/or methods were applied<br><br>**Field Research**<br><br>Include the following details if this study involves the collection of plant, animal, or other materials from a natural setting:<br>• Field permit number<br>• Name of the institution or relevant body that granted permission	
**Data Availability**<br><br>Authors are required to make all data underlying the findings described fully available, without restriction, and from the time of publication. PLOS allows rare exceptions to address legal and ethical concerns. See the PLOS Data Policy and FAQ for detailed information.	Yes - all data are fully available without restriction

A Data Availability Statement describing where the data can be found is required at submission. Your answers to this question constitute the Data Availability Statement and **will be published in the article**, if accepted.<br><br>**Important:** Stating 'data available on request from the author' is not sufficient. If your data are only available upon request, select 'No' for the first question and explain your exceptional situation in the text box.<br><br>Do the authors confirm that all data underlying the findings described in their manuscript are fully available without restriction?	
**Describe where the data may be found in full sentences. If you are copying our sample text, replace any instances of XXX with the appropriate details.**<br><br>• If the data are **held or will be held in a public repository**, include URLs, accession numbers or DOIs. If this information will only be available after acceptance, indicate this by ticking the box below. For example: *All XXX files are available from the XXX database (accession number(s) XXX, XXX.*).<br>• If the data are all contained **within the manuscript and/or Supporting Information files**, enter the following: *All relevant data are within the manuscript and its Supporting Information files.*<br>• If neither of these applies but you are able to provide **details of access elsewhere**, with or without limitations, please do so. For example:<br><br>*Data cannot be shared publicly because of [XXX]. Data are available from the XXX Institutional Data Access / Ethics Committee (contact via XXX) for researchers who meet the criteria for access to confidential data.*<br><br>*The data underlying the results presented in the study are available from (include the name of the third party*	All relevant data are within the manuscript and its Supporting Information files.

and contact information or URL).<br>• This text is appropriate if the data are owned by a third party and authors do not have permission to share the data.<br><br>* typeset	
Additional data availability information:	

Powered by Editorial Manager® and ProduXion Manager® from Aries Systems Corporation

Manuscript Click here to access/download;Manuscript;Manuscript.docx

Feedback perceptions of first year medical residents: A survey study

Rachel Cox[1¶], John Arthur[1¶], Kathryn M Burtson[1]

[1]Department of Internal Medicine, Wright Patterson AFB and Wright State University, Wright-Patterson AFB, Ohio, United States of America

[¶]These authors contributed equally to this work

*Corresponding author

Email: rachel.s.cox5.mil@health.mil

Abstract

Feedback in residency is a necessity for progression toward clinical competency and is included in The Accreditation Council for Graduate Medical Education (ACGME) milestones as an essential component for accreditation. Our study elucidates perceptions of feedback of first-year residents, including confidence in the ability to procure feedback, interest in receiving feedback, effort in procuring feedback, the importance of feedback, and frequency of seeking feedback. Additionally, it aims to identify how these perceptions change after education on building expertise through deliberate practice. First-year internal medicine and neurology residents of a mid-sized university-affiliated residency program answered a five-question 5-point Likert scale questionnaire regarding feedback perceptions before and after attending a workshop about building expertise through effective feedback during residency orientation. Related-Samples Wilcoxon Signed Rank Test was applied for comparing pre versus post-questionnaire data.

Of 31 first-year residents, 29 completed the pre-questionnaire for a completion rate of 93.5%, while 24 of 31 completed the post-questionnaire for a completion rate of 77.4%. Of the five questions, three improved when comparing pre and post responses to, including the questions on confidence in the ability to procure feedback ($p=<0.001$), the effort put into procuring feedback ($p=0.001$), and frequency of seeking feedback ($p=0.002$). Interest in receiving feedback and the importance of feedback remained unchanged after workshop attendance. Residents should be educated on building expertise through deliberate practice and how to obtain high-quality feedback, given the emphasis and essentiality of feedback within the milestone assessment system and the core competencies of ACGME. In our study, education on these topics led to significant improvement in resident perceptions of confidence in the ability to

procure feedback, effort put into procuring feedback, and frequency at which feedback would be sought.

Introduction

In 2013, the ACGME incorporated milestones in trainee assessment as a critical element in the Next Accreditation System. Per “The Milestones Guidebook” by the ACGME in 2020, feedback is an essential and required activity for achieving each milestone within the Six Core Competencies as it is believed feedback is a necessity to achieve clinical competence [1-2]. It is clear that among residency programs, faculty and residents understand just how important feedback is and most residents actively try to change their behavior or practices based on the feedback received [3-5].

Despite this well-known importance, obtaining quality feedback among resident trainees remains variable. Although residents endorse a growth mindset orientation, one survey revealed residents found the act of seeking feedback to induce anxiety and led to subtle tension between supervisors and trainees [6]. In one large study of 17 emergency medicine programs, attending physicians initiated most of the feedback rather than the trainees [7]. Attending physician initiated feedback is suboptimal as feedback is a shared responsibility. Trainees should first set their own goals prior to obtaining feedback as unstructured feedback is general and not specific while regular, specific feedback promotes trainee empowerment [8]. Feedback frameworks such as the ACGME’s Ask-Discuss-Ask-Plan Together model allows for a shared educational alliance that reduces anxiety and promotes continued growth in both the learner and physician [9].

Focused, specific goal setting by trainees and frequent trainee initiated feedback promotes constant progress toward expertise level without falling into automaticity, a concept coined deliberate practice, wherein trainees mindfully push past their comfort zone to achieve

improvement [10]. Deliberate practice was superior to traditional clinical medical education in achieving specific clinical skill acquisition goals; however, deliberate practice requires trainees to identify their weaknesses, something that health professionals have difficulty doing [11-13].

Residents are not empowered to seek feedback as they are often not educated on how to seek regular and specific feedback within their training. There is no current formal or standardized training on resident feedback despite incorporation of feedback into the ACGME required milestones. Education on how to seek quality feedback can improve deliberate practice, feedback behaviors, and professional growth and development.

Materials and Methods

This study was conducted at Wright State University Department of Medicine in a single day during the first-year Wright State resident orientation. Participants consisted of thirty-one incoming 1st-year internal medicine and neurology residents who were required to attend a 90-minute workshop about building expertise through effective feedback, presented by the Wright State Internal Medicine Associate Program Director. Before the workshop, residents anonymously answered a five-question 5-point Likert scale questionnaire that would determine their confidence in the ability to procure feedback, interest in receiving feedback, effort in procuring feedback, the importance of feedback, and frequency of seeking feedback. The questionnaire was sent via a SurveyMonkey link to their mobile devices, where they created their unique username.

The content of the workshop focused on deliberate practice with its components consisting of the following: having specific goals, individualized practice, being outside your comfort zone, and continuous feedback with associated modifications to one’s behaviors as

needed. Furthermore, residents were taught to develop specific, measurable, attainable, realistic, and timely goals. The core learning strategy utilized was the think-pair-share concept to assist residents in formulating their own individualized goals and assessing them for quality allowing for more effective feedback by faculty and staff. This workshop was developed using Kern's 6-step model, including (i) problem identification and general needs assessment, (ii) targeted needs assessment, (iii) goals and objectives, (iv) educational strategies, (v) implementation, and (vi) feedback and evaluation.

Upon completion of the workshop, participants would complete the same questionnaire using their unique username, to determine if there were any changes in their perceptions of feedback. A Related-Samples Wilcoxon Signed Rank Test was selected to compare the pre and post-questionnaire data.

This activity was reviewed by the Wright-Patterson Medical Center Human Research Protection Program (Protocol #: FWP20230001N), and determined to not constitute research following Federal Regulations and Department of Defense Instructions.

Results

Of 31 first-year residents, 29 completed the pre-questionnaire for a completion rate of 93.5%, while 24 of 31 completed the post-questionnaire for a completion rate of 77.4%. Table 1 reveals that on pre to post-questionnaire comparison, using the Related-Samples Wilcoxon Signed Rank Test as described above, three of the five questions show improvement on analysis including the questions regarding confidence in ability to procure feedback with improvement from 3.52±0.57 to 4.37±0.58 ($p<0.001$, ES=0.59), effort put into procuring feedback with improvement from 4.14±0.74 to 4.50±0.59 ($p=0.001$, ES=0.26), and how often feedback is

sought from a faculty member with improvement from 4.34±0.55 to 4.75±0.53 (p=0.002, ES=0.35). In contrast, the questions regarding interest in receiving feedback and the importance of feedback did not change at 4.83±0.38 to 4.83±0.48 (p<0.56) and 4.79±0.49 to 4.87±0.45 (p=0.08) respectively.

Table 1.

	Pre (n= 29)	SD	Post (n= 24)	SD	P value	Effect size
How confident are you in your ability to procure feedback in your residency training?	3.52	0.57	4.37	0.58	<0.001	0.59
How interested are you in receiving feedback throughout your residency training?	4.83	0.38	4.83	0.48	<0.56	N/A
How much effort will you put into procuring feedback during residency training?	4.14	0.74	4.50	0.59	0.001	0.26
How important is feedback to your residency training?	4.79	0.49	4.87	0.45	0.08	N/A
How often will you seek feedback from a faculty	4.34	0.55	4.75	0.53	0.002	0.35

member in your residency training?							

Discussion

Residents are not empowered to seek feedback as they are often not taught how to effectively seek quality feedback in residency. Education on feedback may lead to improvement in feedback behaviors, deliberate practice, and professional growth and development.

Resident perception of confidence in ability to procure feedback improved significantly after education on goal creation and deliberate practice with moderate effect size. Although resident confidence in the setting of feedback has not been examined prior to this study, improvement in confidence may lead to less perceived tension and anxiety surrounding obtaining feedback which are known barriers to residents seeking feedback [6].

Resident effort put forth into obtaining feedback also improved significantly with small effect size. Resident effort as it relates to obtaining feedback has not been investigated prior to this study; however, it has been shown that feedback is usually initiated by attending physicians [7]. Through perceived improvement in effort, residents may feel more empowered to seek feedback after education on feedback and deliberate practice.

Resident perception of frequency of seeking feedback improved significantly with small effect size. In previous investigations, regular, specific feedback led to trainee empowerment and perception of seeking feedback more frequently could promote empowerment and advancement toward expertise rather than suboptimal, general feedback [8].

Perceptions of interest in receiving feedback and importance of feedback showed no change as pre-questionnaire ratings of these variables were high before the workshop and remained high on the post-questionnaire signaling that residents are highly interested and value feedback regardless of formal instruction on the topic.

This study's limitations include that it only examines a single mid-sized university-affiliated residency program, only a single year of residents, and only residents within two specialties. Baseline perceptions of feedback may differ amongst different specialties, residency programs, and resident years which could be viewed as a limitation. Additionally, this quantitative study focuses on level one of the Kirkpatrick Education Model, meaning it examines the participant's reaction to the training. For this reason, it is unknown if residents changed their feedback practices.

Future directions of this study could involve examining residents across different years and specialties and could be extended to additional graduate medical education and undergraduate medical education programs. Qualitative methods, such as semi-structured interviews, could elucidate how feedback behaviors were affected by this workshop. Level three of the Kirkpatrick Education Model could also be examined through the lens of this study, which would include resident change in behavior in feedback practices.

Conclusions

Education on feedback enhances resident perception of confidence, effort, and frequency as it relates to seeking and obtaining feedback in residency and this could lead to higher efficacy and efficiency in reaching ACGME milestones. Residents may feel more empowered to seek feedback after instruction on how to do so effectively. Instruction on feedback as a necessity and

standardization by ACGME of this instruction could be beneficial to residency programs nationwide.

Acknowledgements

We thank Dr. Ronald J. Markert, former vice chair for research in Wright State University Department of Internal Medicine, for statistical analysis and content review of the survey data.

References

1. Edgar L, McLean S, Hogan SO, Hamstra S, Holmboe E. The Milestones Guidebook. 2020: Accreditation Council for Graduate Medical Education; 2020.
2. Ende J. Feedback in clinical medical education. JAMA: The Journal of the American Medical Association. 1983;250(6):777. doi:10.1001/jama.1983.03340060055026
3. Jensen AR, Wright AS, Kim S, Horvath KD, Calhoun KE. Educational feedback in the operating room: A gap between resident and faculty perceptions. The American Journal of Surgery. 2012;204(2):248–55. doi:10.1016/j.amjsurg.2011.08.019
4. Angus S, Moriarty J, Nardino RJ, Chmielewski A, Rosenblum MJ. Internal Medicine residents' perspectives on receiving feedback in milestone format. Journal of Graduate Medical Education. 2015;7(2):220–4. doi:10.4300/jgme-d-14-00446.1
5. Albano S, Quadri SA, Farooqui M, Arangua L, Clark T, Fischberg GM, et al. Resident perspective on feedback and barriers for use as an educational tool. Cureus. 2019; doi:10.7759/cureus.4633
6. Branfield Day L, Miles A, Ginsburg S, Melvin L. Resident perceptions of assessment and feedback in competency-based Medical Education: A FOCUS Group Study of one Internal Medicine Residency Program. Academic Medicine. 2020;95(11):1712–7. doi:10.1097/acm.0000000000003315
7. Yarris LM, Linden JA, Gene Hern H, Lefebvre C, Nestler DM, Fu R, et al. Attending and resident satisfaction with feedback in the emergency department. Academic Emergency Medicine. 2009;16. doi:10.1111/j.1553-2712.2009.00592.x
8. Graffam B, Bowers L, Keene KN. Using observations of clinicians??? teaching practices to build a model of clinical instruction. Academic Medicine. 2008;83(8):768–74. doi:10.1097/acm.0b013e31817ebfa2

9. Fainstad T, McClintock AA, Van der Ridder MJ, Johnston SS, Patton KK. Feedback can be less stressful: Medical trainee perceptions of using the prepare to adapt (ASK-discuss-ask-plan together) framework. Cureus. 2018; doi:10.7759/cureus.3718
10. Ericsson A. Peak: Secrets from the New Science of Expertise. Boston, MA: Random House UK; 2017.
11. Ericsson A. The influence of experience and deliberate practice on the development of superior expert performance. In: The Cambridge Handbook of Expertise and expert performance. Cambridge, United Kingdom: Cambridge University Press; 2006. p. 683–703.
12. 1. Davis DA, Mazmanian PE, Fordis M, Van Harrison R, Thorpe KE, Perrier L. Accuracy of physician self-assessment compared with observed measures of competence. JAMA. 2006;296(9):1094. doi:10.1001/jama.296.9.1094
13. 1. Gordon MJ. A review of the validity and accuracy of self-assessments in health professions training. Academic Medicine. 1991;66(12):762–9. doi:10.1097/00001888-199112000-00012

Click here to access/download
Supporting Information
S1 Dataset.xlsx

Feedback perceptions of first year medical residents: An intervention-based survey study

Rachel Cox[1¶], John Arthur[1¶], Kathryn M Burtson[1]

[1]Department of Internal Medicine, Wright Patterson AFB and Wright State University, Wright-Patterson AFB, Ohio, United States of America

[¶]These authors contributed equally to this work

*Corresponding author

Email: rachel.s.cox5.mil@health.mil

Formatted: Header, Indent: Left: -0.08"

Formatted: Header, Right, Right: -0.08"

Formatted Table

Formatted: Header, Centered

Formatted: Footer

Abstract

Background

Feedback in residency is a necessity for progression toward clinical competency and is included in The Accreditation Council for Graduate Medical Education (ACGME) milestones as an essential component for accreditation.

Purpose

Our study elucidates perceptions of feedback of first-year residents~~, including confidence in the ability to procure feedback, interest in receiving feedback, effort in procuring feedback, the importance of feedback, and frequency of seeking feedback. Additionally, it~~ and aims to identify how these perceptions change after education on building expertise through deliberate practice.

Methods

First-year internal medicine and neurology residents of a mid-sized university-affiliated residency program answered a five-question 5-point ~~Likert~~ unipolar response scale questionnaire regarding feedback perceptions before and after attending a workshop about building expertise through effective feedback during residency orientation. Related-Samples Wilcoxon Signed Rank Test was applied for comparing ~~pre versus post-questionnaire~~pre- versus post-questionnaire data.

Results

Formatted: Font: (Default) Times New Roman, 14 pt, Bold

Formatted: Font: 14 pt

Formatted: Indent: First line: 0"

Formatted: Font: 14 pt, Bold

Formatted: Indent: First line: 0"

Formatted: Font: 14 pt, Bold

Formatted: Indent: First line: 0"

Formatted: Font: 14 pt, Bold

Formatted: Indent: First line: 0"

Formatted: Header, Indent: Left: -0.08"

Formatted: Header, Right, Right: -0.08"

Formatted Table

Formatted: Header, Centered

Formatted: Footer

Of 31 first-year residents, 29 completed the pre-questionnaire for a completion rate of 93.5%, while 24 of 31 completed the post-questionnaire for a completion rate of 77.4%. Of the five questions, three improved when comparing pre and post responses to, including the questions on confidence in the ability to procure feedback (p=<0.001), the effort put into procuring feedback (p=0.001), and frequency of seeking feedback (p=0.002). Interest in receiving feedback and the importance of feedback remained unchanged after workshop attendance.

Conclusion

Residents should be educated on building expertise through deliberate practice and how to obtain high-quality feedback, given the emphasis and essentiality of feedback within the milestone assessment system and the core competencies of ACGME. In our study, education on these topics led to significant improvement in resident perceptions of confidence in the ability to procure feedback, effort put into procuring feedback, and frequency at which feedback would be sought.

Formatted: Font: Bold
Formatted: Indent: First line: 0"
Formatted: Header, Indent: Left: -0.08"
Formatted: Header, Right, Right: -0.08"
Formatted Table
Formatted: Header, Centered
Formatted: Footer

Introduction

In 2013, the ACGME incorporated milestones in trainee assessment as a critical element in the Next Accreditation System. Per “The Milestones Guidebook” by the ACGME in 2020, feedback is an essential and required activity for achieving each milestone within the Six Core Competencies as it is believed feedback is a necessity to achieve clinical competence [1-2]. It is clear that among residency programs, faculty and residents understand just how important feedback is and most residents actively try to change their behavior or practices based on the feedback received [3-5].

Despite this well-known importance, obtaining quality feedback among resident trainees remains variable. Although residents endorse a growth mindset orientation, one survey revealed residents found the act of seeking feedback to induce anxiety and led to subtle tension between supervisors and trainees [6]. In one large study of 17 emergency medicine programs, attending physicians initiated most of the feedback rather than the trainees [7]. Attending ~~physician initiated~~physician-initiated feedback is suboptimal as feedback is a shared responsibility. Trainees should first set their own goals prior to obtaining feedback as unstructured feedback is general and not specific while regular, specific feedback promotes trainee empowerment [8]. Feedback frameworks such as the ACGME’s Ask-Discuss-Ask-Plan Together model allows for a shared educational alliance that reduces anxiety and promotes continued growth in both the learner and physician [9].

Focused, specific goal setting by trainees and frequent trainee initiated feedback promotes constant progress toward expertise level without falling into automaticity, a concept coined deliberate practice, wherein trainees mindfully push past their comfort zone to achieve improvement [10]. Deliberate practice was superior to traditional clinical medical education in

Formatted: Header, Indent: Left: -0.08"

Formatted: Header, Right, Right: -0.08"

Formatted Table

Formatted: Header, Centered

Formatted: Footer

achieving specific clinical skill acquisition goals; however, deliberate practice requires trainees to identify their weaknesses, something that health professionals have difficulty doing [11-13].

Residents are not empowered to seek feedback as they are often not educated on how to seek regular and specific feedback within their training. There is no current formal or standardized training on resident feedback despite incorporation of feedback into the ACGME required milestones. Education on how to seek quality feedback can improve deliberate practice, feedback behaviors, and professional growth and development.

Materials and Methods

This study was conducted at Wright State University Department of Medicine in a single day during the first-year Wright State resident orientation. Participants consisted of thirty-one incoming 1st-year internal medicine and neurology residents who were required to attend a 90-minute workshop about building expertise through effective feedback, presented by the Wright State Internal Medicine Associate Program Director. Before the workshop, residents anonymously answered a five-question 5-point ~~Likert~~unipolar response scale questionnaire that would determine their confidence in the ability to procure feedback, interest in receiving feedback, effort in procuring feedback, the importance of feedback, and frequency of seeking feedback. The questionnaire including the 5-point unipolar response scale was developed using the AMEE guide for educational research, with responses within the scale ~~respresented~~represented nominally as such: 1= never, 2= once in a while, 3= sometimes, 4= often, and 5= almost always. The questionnaire was sent via a SurveyMonkey link to their mobile devices, where they created their unique username. All survey materials were developed and distributed by the Wright State Internal Medicine Program Director.

Formatted: Header, Indent: Left: -0.08"

Formatted: Header, Right, Right: -0.08"

Formatted Table

Formatted: Header, Centered

Formatted: Footer

The content of the workshop focused on deliberate practice with its components consisting of the following: having specific goals, individualized practice, being outside your comfort zone, and continuous feedback with associated modifications to one's behaviors as needed. Furthermore, residents were taught to develop specific, measurable, attainable, realistic, and timely goals. The core learning strategy utilized was the think-pair-share concept to assist residents in formulating their own individualized goals and assessing them for quality allowing for more effective feedback by faculty and staff. This workshop was developed using Kern's 6-step model, including (i) problem identification and general needs assessment, (ii) targeted needs assessment, (iii) goals and objectives, (iv) educational strategies, (v) implementation, and (vi) feedback and evaluation.

Upon completion of the workshop, participants would complete the same questionnaire using their unique username, to determine if there were any changes in their perceptions of feedback. After the conversion of nominal values to scale values, a A Related-Samples Wilcoxon Signed Rank Test was appliedd selected to compare the prepre- and post-questionnaire data.

This activity was reviewed by the Wright-Patterson Medical Center Human Research Protection Program (Protocol #: FWP20230001N), and determined to not constitute research following Federal Regulations and Department of Defense Instructions.

Formatted: Indent: First line: 0.5"

Formatted: Font: (Default) Arial, 11 pt

Results

Of 31 first-year residents, 29 completed the pre-questionnaire for a completion rate of 93.5%, while 24 of 31 completed the post-questionnaire for a completion rate of 77.4%. Table 1

Formatted: Header, Indent: Left: -0.08"

Formatted: Header, Right, Right: -0.08"

Formatted Table

Formatted: Header, Centered

Formatted: Footer

reveals that on pre to post-questionnaire comparison, using the Related-Samples Wilcoxon Signed Rank Test as described above, three of the five questions show improvement on analysis including the questions regarding confidence in ability to procure feedback with improvement from 3.52±0.57 to 4.37±0.58 ($p<0.001$, ES=0.59), effort put into procuring feedback with improvement from 4.14±0.74 to 4.50±0.59 ($p=0.001$, ES=0.26), and how often feedback is sought from a faculty member with improvement from 4.34±0.55 to 4.75±0.53 ($p=0.002$, ES=0.35). In contrast, the questions regarding interest in receiving feedback and the importance of feedback did not change at 4.83±0.38 to 4.83±0.48 ($p<0.56$) and 4.79±0.49 to 4.87±0.45 ($p=0.08$) respectively.

Table 1. Analyses of Pre and Post Feedback Perceptions Questionnaire Responses.

	Pre (n= 29)	SD	Post (n= 24)	SD	P value	Effect size
How confident are you in your ability to procure feedback in your residency training?	3.52	0.57	4.37	0.58	<0.001	0.59
How interested are you in receiving feedback throughout your residency training?	4.83	0.38	4.83	0.48	<0.56	N/A

Formatted: Header, Indent: Left: -0.08"
Formatted: Header, Right, Right: -0.08"
Formatted Table
Formatted: Header, Centered
Formatted: Footer

How much effort will you put into procuring feedback during residency training?	4.14	0.74	4.50	0.59	0.001	0.26
How important is feedback to your residency training?	4.79	0.49	4.87	0.45	0.08	N/A
How often will you seek feedback from a faculty member in your residency training?	4.34	0.55	4.75	0.53	0.002	0.35

Discussion

Residents are not empowered to seek feedback as they are often not taught how to effectively seek quality feedback in residency. Education on feedback may lead to improvement in feedback behaviors, deliberate practice, and professional growth and development.

Resident perception of confidence in ability to procure feedback improved significantly after education on goal creation and deliberate practice with moderate effect size. The content of the workshop contributes to this relaticevelyrelatively larger effect size as residents were educated specifically on how to effectively seek feedback which likely lead to a relatively greater increase in confidence.- Although resident confidence in the setting of feedback has not been examined prior to this study, improvement in confidence may lead to less perceived tension and anxiety surrounding obtaining feedback which are known barriers to residents seeking feedback [6].

Formatted: Header, Indent: Left: -0.08"

Formatted: Header, Right, Right: -0.08"

Formatted Table

Formatted: Header, Centered

Formatted: Footer

Resident effort put forth into obtaining feedback also improved significantly with small effect size. Resident effort as it relates to obtaining feedback has not been investigated prior to this study; however, it has been shown that feedback is usually initiated by attending physicians [7]. Through perceived improvement in effort, residents may feel more empowered to seek feedback after education on feedback and deliberate practice.

Resident perception of frequency of seeking feedback improved significantly with small effect size. In previous investigations, regular, specific feedback led to trainee empowerment and perception of seeking feedback more frequently could promote empowerment and advancement toward expertise rather than suboptimal, general feedback [8].

Perceptions of interest in receiving feedback and importance of feedback showed no change as pre-questionnaire ratings of these variables were high before the workshop and remained high on the post-questionnaire signaling that residents are highly interested and value feedback regardless of formal instruction on the topic.

This study’s limitations include that it only examines a single mid-sized university-affiliated residency program, only a single year of residents, and only residents within two specialties which limits the ability to generalize the results.. Although statistical significance was achieved, the small sample size of this study is a limitation. The results of this study may have been altered by the inclusion of non-first year residents, residents across different disciplines, residents within various sized residency programs, or by inclusion of a greater number of residents in general. The ability to replicate this study is unknown at this time. Baseline perceptions of feedback may differ amongst different specialties, residency programs, and resident years which could be viewed as a limitation

Formatted: Header, Indent: Left: -0.08"

Formatted: Header, Right, Right: -0.08"

Formatted Table

Formatted: Header, Centered

Formatted: Footer

~~.~~Additionally, various trainee characteristics including gender, ethnicity, cultural background, individual learning style, previous experiences and medical school background are not known which is a major limitation and could introduce bias as these characteristics could influence their perception of feedback. Although this study was not designed, approved, or powered to assess the effect of these characteristics on feedback, this would be an important area to include in future studies.

~~Additionally~~Lastly, this quantitative study focuses on level one of the Kirkpatrick Education Model, meaning it examines the participant's reaction to the training. For this reason, it is unknown if residents changed their feedback practices or if this intervention had any contribution to trainee professional ~~devlopment~~development. ~~.~~It is not known whether this intervention lead to any lasting change in perception of feedback or had impact on any other areas of graduate medical education as it pertains to resident growth. Similarly, this study does not investigate specific feedback related outcomes of the workshop to include quality, timeliness, or frequency of feedback sought by trainees.

Future directions of this study could involve examining residents across different years and specialties and could be extended to additional graduate medical education and undergraduate medical education programs. Qualitative methods, such as semi-structured interviews, could elucidate how feedback behaviors were affected by this workshop. Level three of the Kirkpatrick Education Model could also be examined through the lens of this study, which would include resident change in behavior in feedback practices.

Formatted: Indent: First line: 0.5"

Formatted: Header, Indent: Left: -0.08"

Formatted: Header, Right, Right: -0.08"

Formatted Table

Formatted: Header, Centered

Formatted: Footer

Conclusions

Education on feedback enhances resident perception of confidence, effort, and frequency as it relates to seeking and obtaining feedback in residency and this could lead to higher efficacy and efficiency in reaching ACGME milestones. Residents may feel more empowered to seek feedback after instruction on how to do so effectively. Instruction on feedback as a necessity and standardization by ACGME of this instruction could be beneficial to residency programs nationwide.

Acknowledgements

We thank Dr. Ronald J. Markert, former vice chair for research in Wright State University Department of Internal Medicine, for statistical analysis and content review of the survey data.

References

1. Edgar L, McLean S, Hogan SO, Hamstra S, Holmboe E. The Milestones Guidebook. 2020: Accreditation Council for Graduate Medical Education; 2020.
2. Ende J. Feedback in clinical medical education. JAMA: The Journal of the American Medical Association. 1983;250(6):777. doi:10.1001/jama.1983.03340060055026
3. Jensen AR, Wright AS, Kim S, Horvath KD, Calhoun KE. Educational feedback in the operating room: A gap between resident and faculty perceptions. The American Journal of Surgery. 2012;204(2):248–55. doi:10.1016/j.amjsurg.2011.08.019
4. Angus S, Moriarty J, Nardino RJ, Chmielewski A, Rosenblum MJ. Internal Medicine residents' perspectives on receiving feedback in milestone format. Journal of Graduate Medical Education. 2015;7(2):220–4. doi:10.4300/jgme-d-14-00446.1
5. Albano S, Quadri SA, Farooqui M, Arangua L, Clark T, Fischberg GM, et al. Resident perspective on feedback and barriers for use as an educational tool. Cureus. 2019; doi:10.7759/cureus.4633
6. Branfield Day L, Miles A, Ginsburg S, Melvin L. Resident perceptions of assessment and feedback in competency-based Medical Education: A FOCUS Group Study of one Internal Medicine Residency Program. Academic Medicine. 2020;95(11):1712–7. doi:10.1097/acm.0000000000003315
7. Yarris LM, Linden JA, Gene Hern H, Lefebvre C, Nestler DM, Fu R, et al. Attending and resident satisfaction with feedback in the emergency department. Academic Emergency Medicine. 2009;16. doi:10.1111/j.1553-2712.2009.00592.x
8. Graffam B, Bowers L, Keene KN. Using observations of clinicians??? teaching practices to build a model of clinical instruction. Academic Medicine. 2008;83(8):768–74. doi:10.1097/acm.0b013e31817ebfa2
9. Fainstad T, McClintock AA, Van der Ridder MJ, Johnston SS, Patton KK. Feedback can be less stressful: Medical trainee perceptions of using the prepare to adapt (ASK-discuss-ask-plan together) framework. Cureus. 2018; doi:10.7759/cureus.3718
10. Ericsson A. Peak: Secrets from the New Science of Expertise. Boston, MA: Random House UK; 2017.
11. Ericsson A. The influence of experience and deliberate practice on the development of superior expert performance. In: The Cambridge Handbook of Expertise and expert performance. Cambridge, United Kingdom: Cambridge University Press; 2006. p. 683–703.

Formatted: Header, Indent: Left: -0.08"

Formatted: Header, Right, Right: -0.08"

Formatted Table

Formatted: Header, Centered

Formatted: Footer

12. 1. Davis DA, Mazmanian PE, Fordis M, Van Harrison R, Thorpe KE, Perrier L. Accuracy of physician self-assessment compared with observed measures of competence. JAMA. 2006;296(9):1094. doi:10.1001/jama.296.9.1094

13. 1. Gordon MJ. A review of the validity and accuracy of self-assessments in health professions training. Academic Medicine. 1991;66(12):762–9. doi:10.1097/00001888-199112000-00012

Formatted: Header, Indent: Left: -0.08"

Formatted: Header, Right, Right: -0.08"

Formatted Table

Formatted: Header, Centered

Formatted: Footer

~~13.~~ S1 Dataset. Underlying dataset for Pre and Post Feedback Perceptions Responses

Formatted: No bullets or numbering

Formatted: Header, Indent: Left: -0.08"

Formatted: Header, Right, Right: -0.08"

Formatted Table

Formatted: Header, Centered

Formatted: Footer

Reviewer(s)' Comments to Author:

Reviewer: 1

It's a good study looking into the education of trainees, which is often overlooked and has limited interest among researchers. I like the methodology and the results are suggestive to improving the feedback process for trainees. In terms of the results, I would like to know more about the trainees who took the survey. For example, males/ females, IMG/ AMG, ethnicity, etc. Trainees from varying demographics could have a different perception to the feedback process. If this data is not available, it could be a major limitation.

Thank you for the feedback. Demographic information was not included in our IRB proposal or survey instrument. We added to the limitations section to address this and would plan to acquire this data in similar studies in the future.

Reviewer: 2

Title: It is not a simple survey study. This is an intervention-based survey study. Title could be modified a bit to reflect that

We agree that this is not a simple survey study, the title was altered to reflect that.

Materials and Methods: Line 97- It would be helpful for the readers to know how you scored the 5-point likert scale. Especially, how you scored that the last question on how frequently they will seek feedback. Maybe include this scale as a separate figure.

We used the AMEE educational research guide to develop our 5-point unipolar response scales. The nominal values were represented as scale values for each question as such: 1= never, 2= once in a while, 3= sometimes, 4= often, and 5= almost always. The questionnaire was converted from the nominal value to the scale value in order to apply a Related-Samples Wilcoxon Signed Rank Test within SPSS to compare pre- and post-questionnaire data. We have expanded on the Materials and Methods section to expand on the development and analysis of the scale.

Discussion: line 139-143- the relatively bigger effect size of confidence in ability to procure feedback compared to other questions in the survey could be explained by the content of the workshop which was geared specifically to train residents to seek feedback more effectively. This could be discussed more here.

We agree that the relatively larger effect size is likely explained by the context and the content of the workshop. We added to the discussion to recognize that these are contributors to a larger effect size.

Reviewer: 3

1. Authors may consider condensing the length of the abstract as it is somewhat lengthy. Moreover, it is recommended to structure the abstract by providing subheadings for improved clarity. This can be done by organizing the abstract into sections such as Background, Method, Results, and Conclusion.

Some of the abstract was removed to make it more concise. Additionally, subheadings were added for improved organization to include Background, Purpose, Methods, Results, and Conclusion.

2. If the trainees involved in the study come from similar backgrounds or have similar perspectives, this can introduce bias into the research or survey study. How this was addressed in this study?

The demographics of the trainees are not known, and this has been added to the limitations section. This study was not designed, approved, or powered to assess the effect of diversity on perceptions of feedback. This would be an important area to explore in future studies.

3. There are several potential biases and issues that could arise when using Kern's 6-step model in a research or survey study conducted by trainees. Trainees may have limited experience of survey studies, which can lead to issues with study design, data collection. Was this addresses or discussed in limitations?

We have added to the methods section to clarify that all survey materials were developed and distributed by the senior author. Trainees were not involved in the development or distribution of the survey.

4. This study does not explore the factors that may influence residents' perception of feedback, such as their previous experiences, individual learning styles, or cultural background. Understanding these factors could provide a more comprehensive understanding of the limitations and potential biases in the residents' perceptions.

Thank you for the feedback. As above, this has been added to our limitations section.

5. The study only focuses on residents' perceptions of feedback and does not evaluate the effectiveness or impact of the feedback on their learning and professional development. It would be valuable to investigate whether the feedback provided to residents actually leads to meaningful changes in their knowledge, skills, and attitudes.

This study was designed to measure resident perceptions and attitudes regarding feedback. An important area of future study would include assessment of feedback knowledge and behavior.

6. The study only considers one aspect of the feedback process, specifically the residents' reactions to the feedback. It does not explore other important dimensions, such as the quality and timeliness of the feedback, and the follow-up actions taken based on the feedback. Investigating these aspects could provide a more comprehensive understanding of the feedback process in residency programs.

We have added that areas of future study include assessment of feedback knowledge and behaviors.

7. Lastly, this study only focuses on a single year of residents in a single residency program. Generalizing the findings to other programs or different cohorts of residents should be done with caution.

We have expanded on the limitation of this study include the population that was studied, a single residency program and trainees of two specialties across a single year.

Reviewer: 4

LIMITATIONS: The sample size is very small. Also like the author has mentioned that perhaps the results would have been different if the second and third year residents were included too. It would have been helpful to include other disciplines besides just internal medicine and Neurology. Also it would be great to see the impact of size of the program ie small sixed (more cohesive) or big residency program on results of the study.

We have expanded on the limitations section to further explain that this study is limited by the inclusion of only first years at a single mid-sized residency and that the generalizability of our results would be mitigated by this research design.
