# Supplementary material for: Feedback perceptions of first year medical residents: An intervention-based survey study
Source: PLoS One. 2024 Apr 10;19(4):e0300205. doi: 10.1371/journal.pone.0300205 (PMC11006118; doi:10.1371/journal.pone.0300205)
Supplement: Attachment — Submitted filename: Response to Reviewers.docx [file pone.0300205.s003.docx]

***Reviewer(s)’ Comments to Author:***

*Reviewer: 1*

***It's a good study looking into the education of trainees, which is often overlooked and has limited interest among researchers. I like the methodology and the results are suggestive*** ***to improving the feedback process for trainees. In terms of the results, I would like to know more about the trainees who took the survey. For example, males/ females, IMG/ AMG, ethnicity, etc. Trainees from varying demographics could have a different perception to the feedback process. If this data is not available, it could be a major limitation.***

Thank you for the feedback. Demographic information was not included in our IRB proposal or survey instrument. We added to the limitations section to address this and would plan to acquire this data in similar studies in the future.

***Reviewer: 2***

***Title: It is not a simple survey study. This is an intervention-based survey study. Title could be modified a bit to reflect that***

We agree that this is not a simple survey study, the title was altered to reflect that.

***Materials and Methods: Line 97- It would be helpful for the readers to know how you scored the 5-point likert scale. Especially, how you scored that*** ***the last question on how frequently they will seek feedback. Maybe include this scale as a separate figure.***

We used the AMEE educational research guide to develop our 5-point unipolar response scales. The nominal values were represented as scale values for each question as such: 1= never, 2= once in a while, 3= sometimes, 4= often, and 5= almost always. The questionnaire was converted from the nominal value to the scale value in order to apply a Related-Samples Wilcoxon Signed Rank Test within SPSS to compare pre- and post-questionnaire data. We have expanded on the Materials and Methods section to expand on the development and analysis of the scale.

***Discussion: line 139-143- the relatively bigger effect size of confidence in ability to procure feedback compared to other questions in the survey could be explained by the content of the workshop which was geared specifically to train residents to seek feedback more effectively. This could be discussed more here.***

We agree that the relatively larger effect size is likely explained by the context and the content of the workshop. We added to the discussion to recognize that these are contributors to a larger effect size.

***Reviewer: 3***

***1. Authors may consider condensing the length of the abstract as it is somewhat lengthy. Moreover, it is recommended to structure the abstract by providing subheadings for improved clarity. This can be done by organizing the abstract into sections such as Background, Method, Results, and Conclusion.***

Some of the abstract was removed to make it more concise. Additionally, subheadings were added for improved organization to include Background, Purpose, Methods, Results, and Conclusion.

***2. If the trainees involved in the study come from similar backgrounds or have similar perspectives, this can introduce bias into the research or survey study. How*** ***this was addressed in this study?***

The demographics of the trainees are not known, and this has been added to the limitations section. This study was not designed, approved, or powered to assess the effect of diversity on perceptions of feedback. This would be an important area to explore in future studies.

***3. There are several potential biases and issues that could arise when using Kern's 6-step model in a research or survey study conducted by trainees. Trainees may have limited experience of survey studies, which can lead to issues with study*** ***design, data collection. Was*** ***this addresses or discussed in limitations?***

We have added to the methods section to clarify that all survey materials were developed and distributed by the senior author. Trainees were not involved in the development or distribution of the survey.

***4. This study does not explore the factors that may influence residents' perception of feedback, such as their previous experiences, individual learning styles, or cultural background. Understanding these factors could provide a more comprehensive understanding of the limitations and potential biases in the residents' perceptions.***

Thank you for the feedback. As above, this has been added to our limitations section.

***5. The study only focuses on residents' perceptions of feedback and does not evaluate the effectiveness or impact of the feedback on their learning and professional development. It would be valuable to investigate whether the feedback provided to residents actually leads to meaningful changes in their knowledge, skills, and attitudes.***

This study was designed to measure resident perceptions and attitudes regarding feedback. An important area of future study would include assessment of feedback knowledge and behavior.

***6. The study only considers one aspect of the feedback process, specifically the residents' reactions to the feedback. It does not explore other important dimensions, such as the quality and timeliness of the feedback, and the follow-up actions taken based on the feedback. Investigating these aspects could provide a more comprehensive understanding of the feedback process in residency programs.***

We have added that areas of future study include assessment of feedback knowledge and behaviors.

***7. Lastly, this study only focuses on a single year of residents in a single residency program. Generalizing the findings to other programs or different cohorts of residents should be done with caution.***

We have expanded on the limitation of this study include the population that was studied, a single residency program and trainees of two specialties across a single year.

***Reviewer: 4***

***LIMITATIONS: The sample size is very small. Also like the author has mentioned that perhaps the results would have been different if the*** ***second and third year residents were included too. It would have been helpful to include other disciplines besides just internal medicine and Neurology.*** ***Also it would be great to see the impact of size of the program ie small sixed*** ***( more cohesive) or big residency program on results of the study.***

We have expanded on the limitations section to further explain that this study is limited by the inclusion of only first years at a single mid-sized residency and that the generalizability of our results would be mitigated by this research design.
