# Supplementary material for: Feedback perceptions of first year medical residents: An intervention-based survey study
Source: PLoS One. 2024 Apr 10;19(4):e0300205. doi: 10.1371/journal.pone.0300205 (PMC11006118; doi:10.1371/journal.pone.0300205)
Supplement: Attachment — Submitted filename: Response to Reviewers.docx [file pone.0300205.s005.docx]

***Reviewer(s)’ Comments to Author:***

***Reviewer: Academic Editor***

***Thank you for submitting your manuscript. There is a unanimous decision among reviewers that some minor revisions that would be beneficial for the paper. I encourage the authors to review the points including but not limited to the length of the abstract, the confidence interval and the factors influencing the results.***

Thank you for the feedback. The length of the abstract is currently 259 words decreased from 288 in the original version with a known limitation of 300 words per PLOS formatting guidelines. The confidence intervals were generated using Related-Samples Wilcoxon Signed Rank Test via SPSS. The various factors influencing the results and consequent limitations of these factors have been included in the discussion section.

***Reviewer: 5***

***The authors have responded to all the reviewers comments and also formatted and adjusted the manuscript. The authors' responsiveness reflects their commitment to producing a high-quality piece of work. With these commendable revisions, I believe the manuscript is now well-prepared for the next stage of the review process. We agree that this is not a simple survey study, the title was altered to reflect that.***

We appreciate the feedback.

***Reviewer: 6***

***I have had the honor of reviewing the revised version article titled "Feedback perceptions of first year medical residents: An intervention-based survey study" submitted to PLOS ONE for peer review.

The manuscript addresses a very important as well as a critical aspect of medical education that is feedback in residency, which is a core element for clinical competency. The study aligns with ACGME milestones and contributes to the understanding of residents' perceptions and behaviors related to feedback.

The study results have few limitations as identified by the authors, mainly the study focuses on a single mid-sized university-affiliated residency program, a single year of residents, and only two specialties. The sample size is very small with N=24 who completed a post questionnaire. The findings may not be fully generalizable to different specialties, residency programs, or years. The study primarily focuses on level one of the Kirkpatrick Education Model, examining participants' reactions to training. Future research could explore the impact on residents' actual feedback practices (level three) using qualitative methods. To enhance generalizability, consider including residents from different specialties, years, institutions and different demographics. This could provide a broader understanding of how feedback perceptions vary across diverse settings. It looks like all the above-mentioned limitations were raised by the reviewers and the authors made diligent efforts to disclose those in their revised manuscript.***

We appreciate the opportunity to highlight further limitations and direction of future study.

***Reviewer: 7***

***Seems like an interesting study, wonder if there will be longitudinal follow up to ascertain improvement in seeking feedback.
Please see attached file***

A citation was added to highlighted sentence in line 73 as noted in the comment in attached file by Reviewer 7, we appreciate the feedback. We recognize that there is no longitudinal component to this study as noted in the discussion, this is certainly an area for study in the future for follow up on these findings.
